# A Prospective, Observational Study of Adverse Reactions to Drug Regimen for Multi-Drug Resistant Pulmonary Tuberculosis in Central India

**DOI:** 10.4084/MJHID.2014.061

**Published:** 2014-09-01

**Authors:** Rohan Hire, A. S. Kale, G. N. Dakhale, Nilesh Gaikwad

**Affiliations:** Department of Pharmacology, Government Medical College, Nagpur, India-440003

## Abstract

**Objective:**

1) To assess the adverse drug reactions (ADRs) of second-line anti-tubercular drugs used to treat Multi-drug resistant Tuberculosis (MDR-TB) in central India on the basis of causality, severity and avoidability scales. 2) To investigate the relationship of MDR-TB (primary or secondary) and the presence of diabetes mellitus (DM) with mean smear conversion time. *Material and Methods:* A prospective, observational study was carried out on diagnosed multidrug-resistant tuberculosis patients enrolled for DOTS-Plus regimen at TB and Chest Disease Department from January 2012 to December 2012 with a follow-up of nine months. Encountered ADRs were noted along with the time of sputum conversion.

**Results:**

Total 64 ADRs were reported in 55 patients out of total 110 patients (n=110). As per the Naranjo causality assessment of ADRs, seven patients had definite, 45 had probable, and 3 had possible causal relation with drugs of DOTS-Plus regimen. As per the Hartwig’s severity assessment scale, there were total 7 ADRs in Level 1, 6 in Level 2, 33 in Level 3 and 9 in Level 4. Hallas avoidability assessment scale divided the ADRs as 3 being definitely avoidable, 26 possibly avoidable, 23 not avoidable and three not evaluable. Mean sputum smear conversion time was significantly higher in patients with a secondary type than that of primary type of MDR TB and in patients with DM than those without DM.

**Conclusion:**

ADRs were common in patients of MDR-TB on DOTs-Plus drug regimen. It was due to lack of availability of safer and equally potent drugs in DOTs-Plus drug regimen compared to DOTS regimen in non-resistant TB. The frequency and severity of ADRs can be reduced by strict vigilance about known and unknown ADRs, monitoring their laboratory and clinical parameters and instituting appropriate measures.

## Introduction

World Health Organization Global TB Report 2011 has reported an estimated 8.8 million incident cases of TB globally in 2010 and 1.1 million deaths among HIV-negative cases of TB. WHO has ranked TB as the seventh most morbidity-causing disease in the world. Out of the estimated global annual incidence of 9.4 million TB cases, two million have been estimated in India, thus contributing to a fifth of the global burden of TB.^1^ Today, the major problem is the emergence of multi-drug resistant (MDR) and extensively drug-resistant (XDR) TB. Drug resistance surveys, based on state representative community, were carried out in the states of Gujarat, Maharashtra and Andhra Pradesh. The prevalence of MDR-TB was estimated to be 3%, among new TB cases and 12–17%, among previously-treated TB cases.^2^ MDR-TB is important as patients with this type of drug resistance respond extremely poorly to standard anti-TB treatment with first-line drugs.^3^ Pilot studies have shown that DOTS has been successful in reducing the prevalence of drug-resistant TB on a community level in Mexico, Peru, and India.

The treatment of MDR-TB is of longer duration for about two years. Therefore, it is imperative to monitor and treat adverse drug reactions developed by the patients. This approach ensures better compliance of patients and good treatment outcome. At the same time, data regarding ADRs of second-line anti-tubercular drugs in Central India are scanty. Hence, the aim of this study was to assess the adverse drug reactions of second-line anti-tubercular drugs used to treat MDR-TB in central India on the basis of causality, severity and avoidability scales. At the same time, we studied the relationship between MDR-TB type and presence of diabetes mellitus (DM) with mean smear conversion time.

## Materials and Methods

It was a prospective observational study of assessing adverse reactions to drug regimen for multi-drug resistant tuberculosis. This study was carried out at TB and Chest Disease Department of Government medical college, Nagpur. The duration of the study was one year. Diagnosed case of MDR-TB is defined as an MDR-TB suspect who is sputum culture positive and whose TB is due to Mycobacterium tuberculosis that are resistant in-vitro to at least isoniazid and rifampicin.^3^ They were put on MDR-TB drug regimen also called as DOTS-Plus regimen or category IV regimen. Approval of Institutional human research ethics committee was obtained and written informed consent of enrolled patients was taken before conducting the study. Diagnosed patients of multi-drug resistant pulmonary tuberculosis above 18 years of age, who were having all pre-treatment investigations normal, were included in the study. Patients under 18 years of age, pregnant women, patients, HIV-positive, or having concurrent major psychiatric illness and/or concurrent major medical illnesses, or having had more than one month treatment with any second line anti-tubercular drugs were excluded from the study.

The patients were monitored for the period of nine months from the day of commencement of treatment. The diagnosis of MDR-TB was confirmed by drug sensitivity test prior to enrolment. Before the patients were started on DOTS-Plus regimen, they were submitted to some pretreatment investigation such as, sputum smear, liver function tests, kidney function tests (blood urea, serum creatinine), thyroid function tests, blood sugar levels (fasting and post-prandial), psychiatric screening, HIV seropositivity test and chest X-ray. The intensive phase of DOTS-Plus regimen contain Kanamycin(inj.), Levofloxacin, Ethionamide, Pyrazinamide, Ethambutol, Cycloserine (total six drugs) and continuation phase contain Levofloxacin, Ethionamide, Ethambutol, Cycloserine (total four drugs).

All drugs were given in a single daily dosage under directly observed treatment (DOT) by a DOT Provider. All patients received drugs for six days of the week. On the seventh day (Sunday) the oral drugs were administered unsupervised whereas injection kanamycin was omitted. If intolerance occurred to the drugs, ethionamide, cycloserine and PAS were split into two dosages and the morning dose administered under DOT. The evening dose was self-administered. Doses of the drugs were chosen according the weight range to which patient belonged. Patients were monitored for adverse drug reactions at regular intervals. Initially, they were monitored daily after starting regimen till the patients remains admitted. Once discharged, patients were monitored and followed up on a monthly basis for nine months. Patients were also monitored for any of their complaints. During subsequent visits biochemical investigations were repeated. Chest X-ray was done when required. Patients with severe adverse drug reactions were referred to concerned clinical departments and followed up regularly. Causality assessment was done using Naranjo’s causality assessment scale.^4^ Severity of ADRs was assessed using Hartwig’s severity assessment levels.^5^ Avoidability of ADRs was assessed by using Hallas’s avoidability assessment categories.^6^

### Calculation of sample size and statistical analysis

One hundred and ten patients of diagnosed cases of MDR-TB were enrolled as per the previous studies.^7^ Continuous variables were expressed as mean+SD whereas categorical variables were expressed in absolute numbers or percentages and compared by Chi-square or Fisher’s exact test. Mean sputum smear conversion time was compared with different types of MDR-TB and presence of DM by unpaired t-test. P values less than 0.05 were considered as statistically significant. Statistical software STATA version 10.0 was used for statistical analysis.

## Observation and Results

Out of the total 110 patients, 40 patients belonged to age group 40–49 years while two patients to age group 70–79 years. The age wise incidence of ADRs showed a maximum rate of 17.3 % in 40–49 years and a minimum one of 0.8 % in 70–79 years. There were 83 males and 27 females. Forty out of 83 males and 15 out of 27 females were having at least one ADR. Incidence of ADRs in primary MDR-TB was 16.6 % while in secondary MDR-TB was 51.9 % (Table 1). Incidence of ADRs was more in patients with diabetes mellitus (80%) than in patients without diabetes mellitus (47%) (Table 2). Total 64 ADRs were reported in 55 patients. Gastrointestinal symptoms like nausea and vomiting contributed to 30% of total ADRs (Table 3).

ADRs were categorized in four groups i.e. definite, probable, possible and unlikely based on Naranjo’s causality assessment questionnaire (Table 4). As per causality assessment of ADRs, 7 had definite, 45 probable and 3 had possible causal relation with drugs of DOTS-Plus regimen. These patients were categorised on the basis of a number of ADRs reported i.e. 1, 2, 3. In 48 patients only one ADR, in five patients two ADRs and two patients three ADRs were reported. There were total 7 ADRs in Level 1, 6 in Level 2, 33 in Level 3 and 9 in Level 4 by using Hartwig’s severity assessment scale (Table 5). Maximum numbers of ADRs belonged to Level 3 of severity assessment. Using Hallas avoidability assessment scale, all ADRs are divided into four groups of definitely avoidable, possibly avoidable, not avoidable and not evaluable. Twenty-six ADRs were possibly avoidable, and 23 were not avoidable (Table 6). Maximum sputum smear conversion occurred at the end of the fourth month (42.7 %) and fifth month (37.3 %) while five patients took more than six months for the same. Mean sputum smear conversion time was significantly higher (p=0.0001) in patients with a secondary type (4.62±0.87) than that of primary type (3.16 ±0.41). Mean sputum smear conversion time was significantly higher (p <0.0001) in patients with DM (6.3 ± 0.94) than those without DM (4.62 ± 0.87).

## Discussion

In this study, out of 110 patients enrolled; 55 patients developed at least one to maximum three ADRs. The overall pattern of adverse reactions in our study was comparable to previous studies.^7^,^8^,^9^ Gastrointestinal symptoms (nausea, vomiting and mild gastritis) with severity level 3 were the most-common adverse reactions in our study similar to other findings.^7^,^10^,^11^ Quinolones and ethionamide were found to have probable causal relationship with GI symptoms. They were mild but required immediate treatment. These gastrointestinal symptoms occurred mostly within a week of starting treatment. No patient required alteration in DOTS-Plus treatment due to gastrointestinal ADRs. Quinolones and ethionamide were found to be culprit drugs after causality assessment. It is possible to avoid these ADRs by administering these drugs one hour after one tablet ofdomperidone and proton pump inhibitor or H2 receptor inhibitor. Arthralgia and psychosis were next most common ADRs. Arthralgia with severity level 3 showed probable causal relationship with pyrazinamide and quinolones. Mostly, weight bearing joints were affected within fifteen days of starting the treatment. Pyrazinamide produces arthralgia and arthritis by increasing serum uric acid levels while quinolones cause cartilage toxicity.^12^ Being unpredictable, arthralgia was also not avoidable. Cycloserine was found to have definite causal relationship with psychosis as in previous studies.^13^ It occurred 4–6 months after treatment commencement. Occurrence of psychosis was an unpredictable adverse event and grouped as not avoidable ADR with severity level 4. These patients were started with PAS after omitting cycloserine from DOTS-Plus regimen.

Hepatotoxicity is defined as 1.5 times rise in pre-treatment alanine transaminase (ALT) levels. Pyrazinamide induced hepatotoxicity has probable causal relationship. As per assessment, hepatotoxicity belonged to not avoidable category with severity level 4. More commonly, anti-TB drugs induced hepatotoxicity is due to the formation or reduced clearance of toxic metabolites with derangement of laboratory parameters within two to three months. Recent evidence indicates that drug dosage play an important role in hepatotoxicity facilitated by genetic factors, or polymorphism of drug metabolizing enzymes.^14^ Localised erythematous rash with severity level 1 occurred within seven days to two months of starting DOTS-Plus treatment. Pyrazinamide and quinolones had probable causal relationship. Incidence of rash due to pyrazinamide ranged from 0.1–5 %.^15^,^16^,^17^ The most frequent adverse dermatological effects of pyrazinamide are burning sensation, hypersensitivity dermatitis and photoallergy.^18^ But in our study, we found mild erythematous rashes which did not require alteration in drug regimen. However this ADR, being unpredictable, became not avoidable. Peripheral neuropathy predominantly of sensory type was developed over the period of seven months of initiation of drug treatment.^19^ Ethionamide was found to have probable causal relationship. As ethionamide is structurally related to isoniazid, it interferes with the utilization of pyridoxine (vitamin B6) and its increased excretion in urine may be responsible for this ADR. Neuropathy was treated by additional 100 mg dose of pyridoxine without any alteration in MDR-TB treatment. Afterward, the increasing the dose of pyridoxine from the beginning of therapy made this mild level 1 ADR avoidable.

Renal impairment with severity level 2 was detected by monthly renal function tests (serum urea and creatinine rise over 150 mg/dl and 90 mg/dl respectively) done for first three months of starting treatment. Non-oliguric or polyuric presentation was quite common.^20^ Kanamycin had probable causal relationship. Aminoglycosides firstly cause tubular cytotoxicity by apoptosis and necrosis of these cells; that is followed by reduced glomerular filtration, induced by vascular and mesangial contraction.^21^ Menorrhagia was reported in two female patients within three months of starting drug treatment. It was a rare ADR of ethionamide with possible causal relationship. It may be due to a disturbance in vitamin B6 complex activation in liver that could have caused an alteration in oestrogen-androgen metabolism.^22^ Due to conflicting available evidence, this level 1 ADR was categorized as not valuable. Vertigo was having definite causal relationship with kanamycin, which as all aminoglycosides is known to be vestibulotoxic with level 2 severity.^2^

At present, there is substantial evidence suggesting that this form of neurotoxicity is partly due to the production of free radicals. It is noteworthy that aminoglycosides induce an increase of free radicals either by stimulating the N-methyl-D-aspartate (NMDA) receptor or by the binding to iron.^24^ Consequently the hair cells of the inner ear are damaged and this alteration causes vertigo. Although kanamycin ototoxicity is well known, it is not avoidable, because of the lack of other drugs equally effective

Visual disturbance was found in one patient at the end of one month. It had definite causal relationship with ethambutol with severity level 2. Animal studies have shown ethambutol to deplete zinc from the optic nerve.^25^ Some studies suggested that the toxicity is mediated through an excitotoxic pathway, so that the ganglion cells are rendered sensitive to normal levels of extracellular glutamate.^26^ As it was an unpredictable event in the course of a complete DOTS-Plus treatment, this ADR was categorized as not avoidable. Tendinitis was one of the rare adverse effects of fluoroquinolones (FQ) reported in one of our patients at the end of three months of treatment. It was having possible causal relationship. The exact mechanism of FQ associated tendinopathy remains to be investigated. FQs act by inhibition of bacterial DNA gyrase (topoisomerase II) which is directly involved in DNA replication and cell division.^27^ Animal studies have demonstrated disorganization of the extracellular matrix (ECM) and degenerative changes in tendon cells in fluoroquinolone-treated rats.^28^,^29^ FQ exert a number of effects at cellular level, including reduced expression of some ECM proteins, non-cytotoxic inhibition of tendon cell proliferation and inhibition of tendon cell migration.^30^ Due to lack of sufficient literature, this level 1 ADR was categorized as not valuable. Hypothyroidism was seen in one male patient at the end of the fourth month of initiating drug therapy. The offending drug for hypothyroidism having level 3 severity was ethionamide with probable causal relationship. Ethionamide, being similar in the structure to other thioamides, such as propylthiouracil and methimazole, could inhibit thyroid hormone synthesis through an analogue mechanism of inhibition of iodine organification.^31^ As it was an unpredictable event in the course of a complete DOTS-Plus treatment, this ADR was categorized as not avoidable.

The most widely accepted measure of treatment response in patients with pulmonary TB is the disappearance of acid-fast bacilli (AFB) from both sputum smear and culture. Mean sputum smear conversion time is significantly longer in patients with a secondary type, and also significantly longer in patients suffering from diabetes (DM). These findings are in parallel to the previous studies.^32^,^33^,^34^ In DM, absorption and metabolism of anti-TB drugs may be impaired which may account for delayed sputum smear conversion.^35^ Also, the incidence of ADRs is more in patients with DM than those without DM. All patients were on regular oral hypoglycaemic agents with controlled blood sugar levels and without any history of ADRs to oral hypoglycaemic agents. These patients of DM when started on DOTS-Plus regimen developed ADRs like gastrointestinal symptoms, rash. This may suggest a causal relationship of drugs from DOTS-Plus regimen with these ADRs. However, the ADRs to oral hypoglycaemic agents cannot be completely ruled out. More studies are needed in patients of MDR-TB with DM to establish drug interactions between DOTS-Plus regimen and oral hypoglycaemic agents.

To the best of our knowledge, this is the first report related to exclusive assessment of ADRs of drug regimen for MDR-TB in the population of Central India where presently no such database of ADRs is available. Although the Naranjo scale does not address the main points that are necessary for causality evaluation of potential drug interactions, and the study was of one-year duration with small sample size, the value of its result cannot be ignored. However, large-scale observational study with larger sample size along with longer follow-up period could have given better rate of incidence database for MDR-TB drug regimen associated ADRs.

## Conclusions

It was found that ADRs were common in patients of MDR-TB on DOTs-Plus drug regimen. It was due to lack of availability of safer and equally potent drugs in DOTs-Plus drug regimen compared to DOTS regimen in non-resistant TB. Hence, only vigilance about known and unknown ADRs, assessment of their causality and prompt management can mitigate toxicity. The frequency and severity of known ADRs can be reduced by monitoring laboratory and clinical parameters and instituting appropriate measures. This may help improving the compliance and ultimately quality of patient care. Thus, close monitoring and timely management are essential for treatment adherence and finally to improve outcome in MDR-TB.

## Figures and Tables

**Table 1:** Incidence of ADRs in different types of MDR-TB.

Mdr-Tb	Number of patients	No. of patients with ADRs	No. of patients without ADRs	% of patients with ADRs
Primary	6	1	5	16.6
Secondary	104	54	50	51.9
TOTAL	110	55	55	50.0
p- value		0.2057, ns		

n = 110, ADRs = adverse drug reactions, MDR-TB = multidrug resistant tuberculosis. Data is analyzed by Fisher’s exact test. ns = non significant.

**Table 2:** Incidence of ADRs in patients of MDR-TB with or without DM.

Presence of DM with MDR-TB	Number of patients	No. of patients with ADRs	No. of patients without ADRs	% of patients with ADRs
Yes	10	8	2	80
No	100	47	53	47
TOTAL	110	55	55	48.2
p- value		0.0933, ns		

ADRs = adverse drug reactions, MDR-TB = multidrug resistant tuberculosis, DM = diabetes mellitus. Data were analyzed by Fisher’s exact test. ns = non significant.

**Table 3:** Frequency of individual ADRs noted during treatment of MDR-TB patients**.**

ADR features	No. of patients with ADR	% of each ADR
GI Symptoms	33	30
Arthralgia	5	4.5
Psychosis	5	4.5
Hepatotoxicity	4	3.6
Rash	4	3.6
Peripheral Neuropathy	3	2.7
Renal Impairment	3	2.7
Menstrual irregularities	2	1.8
Vertigo	2	1.8
Visual disturbances	1	0.9
Tendinitis	1	0.9
Hypothyroidism	1	0.9

n = 110, ADRs = adverse drug reactions, MDR-TB = multidrug resistant tuberculosis, GI symptoms = gastrointestinal symptoms.

**Table 4:** Categorization of ADRs by using Naranjo’s causality assessment scale.

No. of ADRs (No. of patients)	Categorization by causality assessment			
	Definite	Probable	Possible	Unlikely
1 (48)	7(14.5)	38(79.16)	3(6.25)	0
2 (5)	0	5(100)	0	0
3 (2)	0	2	0	0

n = 110, ADRs = adverse drug reactions.

**Table 5:** Categorization of ADRs by using Hartwig’s severity assessment levels.

No. of ADRs (No. of patients)	Categorization by severity assessment scale						
	Level 1	Level 2	Level 3	Level 4	Level 5	Level 6	Level 7
1 (48)	7(14.58)	6(12.5)	30(62.5)	5(10.4)	0	0	0
2 (5)	0	0	3(60)	2(40)	0	0	0
3 (2)	0	0	0	2(100)	0	0	0

n = 110, ADRs = adverse drug reactions.

**Table 6:** Categorization of ADRs by Hallas’s avoidability assessment categories.

No. of ADRs (No. of patients)	Categorization by avoidability assessment scale			
	Definitely avoidable	Possibly avoidable	Not avoidable	Not valuable
1 (48)	3(6.25)	26(54.16)	16(33.33)	3(6.25)
2 (5)	0	0	5(100)	0
3 (2)	0	0	2(100)	0

n = 110, ADRs = adverse drug reactions.
